# The Pneumococcal Polysaccharide-Tetanus Toxin Native C-Fragment Conjugate Vaccine: The Carrier Effect and Immunogenicity

**DOI:** 10.1155/2020/9596129

**Published:** 2020-07-04

**Authors:** Rui Yu, Junjie Xu, Tao Hu, Wei Chen

**Affiliations:** ^1^Laboratory of Vaccine and Antibody Engineering, Beijing Institute of Biotechnology, Beijing 100071, China; ^2^State Key Laboratory of Biochemical Engineering, Institute of Process Engineering, Chinese Academy of Sciences, Beijing 100190, China

## Abstract

The encapsulated bacteria, as *Streptococcus pneumonia*, *Haemophilus influenzae* type b, and *Neisseria meningitidis*, cause serious morbidity and mortality worldwide. The capsular polysaccharide (PS), which could elicit a weak T cell-independent immune response, is a vital virulence determinant. One of the strategies to improve the PS-specific immunogenicity is to conjugate PS with a nontoxic carrier protein. Tetanus toxoid (TT) and CRM_197_ are the typical carrier proteins for the PS conjugate vaccines. TT is the inactivated tetanus toxin manipulated with formaldehyde, which suffers from the pollution from residual formaldehyde and the incomplete detoxification. CRM_197_ has the disadvantage of low-yield purification with the requirement of sophisticated culture conditions. Thus, a novel carrier protein without these disadvantages is highly required. The tetanus toxin native C-fragment (Hc) is safe, low-cost, and highly immunogenic with easy purification, which can act as a promising carrier protein. Pneumococcal serogroups 14 and 23F were major epidemic causes of pneumococcal infections. In the present study, the capsular PSs (PS14 and PS23F) were conjugated with Hc, TT, and CRM_197_, respectively. TT- and CRM_197_-based conjugates acted as controls for Hc-based conjugates (PS14-Hc and PS23F-Hc). The structural properties of Hc were not fundamentally changed after conjugated with PS. PS14-Hc and PS23F-Hc could potentiate sound PS-specific antibody levels comparable to the controls. Thus, Hc exhibited a practical carrier effect to help the pneumococcal conjugate vaccines perform good immunogenicities.

## 1. Introduction

The encapsulated bacteria with capsular polysaccharide (PS), as *Streptococcus pneumonia*, *Haemophilus influenzae* type b, and *Neisseria meningitidis*, cause serious morbidity and mortality worldwide [1]. Due to the increasing bacterial resistance to antibiotics, effective vaccinations are urgently required to protect from the encapsulated bacteria-caused diseases [2, 3]. Capsular PS is an important virulence determinant of the encapsulated bacteria and has been used as an antigen for preventive vaccine development [4, 5]. However, the PS vaccines elicit weak T cell-independent immune responses without long-term immunological memories, particularly in children and the old [6, 7].

The conjugate vaccines have been produced by covalent conjugation of the antigenic PS with a nontoxic carrier protein to enhance the immunogenicity of PS vaccines [8–10]. After priming with the carrier protein, the immune response of PS improved by increasing the number of T lymphocytes [11, 12]. The carrier protein can also anchor PS to the B cell MHC-II, which makes the carbohydrate moiety present to T cell and eliciting T cell-dependent response to PS [13, 14]. Thus, the conjugate vaccines have been demonstrated to be immunogenic and capable of inducing immunological memory and high avidity [15].

Tetanus toxoid (TT) is one of the most commonly used carrier proteins, and TT-based conjugate vaccines have been developed to confer protection against meningococcal and pneumococcal diseases [16]. However, TT is derived from *Clostridium tetani* and inactivated with formaldehyde, which suffers from residual toxic formaldehyde [17]. *C. tetani* can resist the inactivation of formaldehyde by forming spores, so it cannot guarantee that TT is completely nontoxic [17]. Besides, TT-based conjugate vaccine showed high rates of irritability, crying, and fever.

Cross-reactive material 197 (CRM_197_) is a carrier protein used in approved conjugate vaccines, such as Prevnar (Pfizer) against *S. pneumonia* [13]. It does not require detoxification with formaldehyde, thus preserves T-helper epitopes and provides more lysyl side chains for conjugation [18]. However, CRM_197_ is classically purified from the culture supernatant of *C. diphtheriae* C7 (*β*197) strain, which suffers from low yield and requires sophisticated culture conditions [19]. Thus, a candidate carrier protein without the disadvantages of the two proteins is urgently needed for the conjugate vaccines.

The native C-fragment of tetanus toxin (Hc) is a well-defined protein that retains many properties such as low allergenicity, low toxicity, binding activities to gangliosides, and immunogenic potency [20]. Due to its advantages in production, characterization, and homogeneity, Hc is a good substitute for TT in the production of tetanus antitoxin [21, 22]. Moreover, Hc could be purified with high yield by a three-step purification based on affinity chromatography.

Pneumococcal serogroups 14 and 23F have been proved to be a leading cause of pneumococcal infections, and their capsular PSs (PS14 and PS23F) are essential virulence determinants [23, 24]. In the present study, PS14 and PS23F were conjugated with Hc, TT, and CRM_197_, respectively. The carrier effect of Hc to enhance the immunogenicity of the conjugate pneumococcal vaccines was thus evaluated. TT-based and CRM_197_-based conjugates acted as controls for Hc-based ones (PS14-Hc and PS23F-Hc). PS14-Hc and PS23F-Hc were both proved to potentiate a strong PS-specific humoral immunity. The biophysical and immunological properties of PS14-Hc and PS23F-Hc were investigated. The study could rationalize the carrier effect of Hc to enhance the immunogenicity of the conjugate pneumococcal vaccines.

## 2. Materials and Methods

### 2.1. Materials and Animals

2-iminothiolane (IT), bovine serum albumin (BSA), 5,5′-dithio-bis-(2-nitrobenzoic acid), and *N*-(2-aminoethyl) maleimide (AM) were purchased from Sigma (USA). Horseradish peroxidase- (HRP-) conjugated goat anti-mouse antibodies (including HRP-IgM, HRP-IgG, HRP-IgG1, and HRP-IgG2a) were products of Abcam (USA). Pneumococcal PS serogroups 14 and 23F, CRM_197_, and TT were products of Hualan Biological Engineering Inc. (China). Hc was prepared as described previously [20].

BALB/c (15-20 g, female) were purchased from Beijing Vital River Laboratory Animal Technologies Co. Ltd. The mice were provided with enough food and water and raised under a comfortable environment. The mouse experiments were approved by the Institutional Animal Care and Use Committee of Beijing Laboratory Animal Research Center (identification code: ZSBD-2017-A034-3R). All operations performed on mice were according to approved methods. The mice were euthanized at the end of the experiment, and all efforts were made to reduce the pain.

### 2.2. Activation of PS

**Figure 1:**
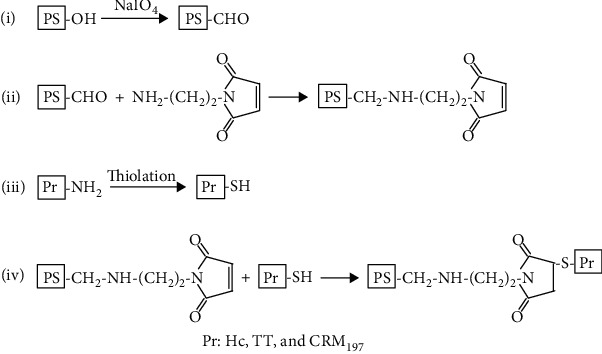
Schematic representation of the conjugates. PS was introduced with maleimide groups by incubation with AM. The carrier proteins were thiolated and reacted with the activated PS.

4 mg/ml PS14 or PS23F with a volume of 20 ml was added to the 20 mM acetate buffer (pH 5.8), which contained 10 mM sodium periodate to conduct an oxidation reaction for 40 minutes (Figure 1). The oxidized PS14 or PS23F with a concentration of 3 mg/ml and a volume of 20 ml, NaCNBH_3_ with a concentration of 10 mg/ml and a volume of 2 ml, and AM with a concentration of 6 mg/ml and a volume of 10 ml were mixed and incubated in 20 mM phosphate buffer (PB buffer, pH 7.4) at 2 ~ 8°C overnight. The activated PS was subsequently dialyzed to 20 mM PB buffer (pH 7.4).

### 2.3. Preparation of PS14-Hc and PS23F-Hc

Hc with a concentration of 3 mg/ml and a volume of 6 ml was added into 20 mM PB buffer (pH 7.4) containing 36 nmol of IT. After being incubated at 2 ~ 8°C overnight, the free IT was removed by extensive dialysis using 20 mM PB buffer (pH 7.4). The activated PS14 (2 mg/ml, 4.5 ml) or PS23F (2 mg/ml, 4.5 ml) was incubated with the resultant Hc derivative at 2 ~ 8°C overnight, respectively. The resultant conjugates were referred to as PS14-Hc and PS23F-Hc, respectively.

### 2.4. Preparation of PS14-TT and PS23F-TT

TT with a concentration of 3 mg/ml and a volume of 9 ml was added into 20 mM PB buffer (pH 7.4) containing 11 nmol of IT. After being incubated at 2 ~ 8°C overnight, the free IT was removed by extensive dialysis using 20 mM PB buffer (pH 7.4). The activated PS14 (2 mg/ml, 4.5 ml) or PS23F (2 mg/ml, 4.5 ml) was incubated with the resultant Hc derivative at 2 ~ 8°C overnight, respectively. PS14-TT and PS23F-TT were referred to as the resultant conjugates, respectively.

### 2.5. Preparation of PS14-CRM and PS23F-CRM

CRM_197_ with a concentration of 3 mg/ml and a volume of 6 ml was added into 20 mM PB buffer (pH 7.4) containing 16 nmol of IT. After being incubated at 2 ~ 8°C overnight, the free IT was removed by extensive dialysis using 20 mM PB buffer (pH 7.4). The activated PS14 (2 mg/ml, 4.5 ml) or PS23F (2 mg/ml, 4.5 ml) was incubated with the resultant Hc derivative at 2 ~ 8°C overnight, respectively. PS14-CRM and PS23F-CRM were referred to as the resultant conjugates, respectively.

### 2.6. Purification of the Conjugates

The purification of the six conjugates was performed by a Superdex 200 column (1.6 cm × 60 cm, GE Healthcare, USA) with a method of size exclusion chromatography (SEC). The column was equilibrated and eluted by 20 mM PB buffer (pH 7.4) with a flow rate of 2.0 ml/min. Then, the target conjugates were collected.

### 2.7. Quantitative Assay

The phenol-sulfuric acid method was used to determine the total PS contents of the conjugates. A quantitative method for the determination of unconjugated PS in conjugates was established. The method was based on the ethanol precipitation of TT. The thiolation degree of TT was detected by 5,5′-dithiobis (2-nitrobenzoic acid). Using BSA as the standard, TT contents in the conjugates were determined by bicinchoninic acid. Then, the mass ratios of PS/TT of the conjugate vaccines were obtained.

### 2.8. Dynamic Light Scattering

Using a Wyatt DynaPro Titan TC instrument (Santa Barbara, CA, USA), the molecular radii of the six conjugates were calculated at 25°C. Before the analysis, the samples were centrifuged at 12,000 g for 10 min in 20 mM PB buffer (pH 7.4) with a concentration of 1 mg/ml.

### 2.9. Intrinsic Fluorescence Spectroscopy

The conjugates were prepared at a protein concentration of 0.15 mg/ml in 20 mM PB buffer (pH 7.4). Using a fluorescence spectropolarimeter (Hitachi, Japan), the intrinsic fluorescence spectra of the six conjugates were recorded. The emission fluorescence intensities were between 300 nm and 400 nm with excitation at 280 nm. The emission and excitation slit widths were both 2.5 nm.

### 2.10. Circular Dichroism Spectroscopy

Far-UV circular dichroism (CD) spectra of the six conjugates were detected by the spectropolarimeter (Jasco, Japan) with the wavelengths from 260 nm to 190 nm. The length of cuvettes used in this detection was 0.2 cm path. All the protein concentrations of the conjugates in 20 mM PB buffer (pH 7.4) were 0.15 mg/ml.

### 2.11. Immunization

BALB/c mice (female, six weeks old) were randomly divided into eight groups with the name of PS14, PS23F, PS14-TT, PS23F-TT, PS14-Hc, PS23F-Hc, PS14-CRM, and PS23F-CRM, respectively. Each group has six mice. The mice were intraperitoneally injected with different vaccines on days 0, 14, and 28, respectively. All the vaccines contained 2 *μ*g/ml of PS in a volume of 0.5 ml. The tail vein blood of mice was taken on days 14, 28, and 42. The sera of mice were stored at -20°C after separation.

### 2.12. Antibody Titers

The anti-PS antibody titers of IgG, IgG1, IgG2a, and IgM were determined by ELISA assay [25]. Briefly, PS14-BSA or PS23F-BSA (2 *μ*g PS/ml) was prepared as the previous description [26] and used to coat the 96-well plates 100 *μ*l per well at 2 ~ 8°C overnight. The plates were washed four times with PBS plus 0.1% Tween 20 (PBST). The serum serially 2-fold diluted from 1 : 100 was added to the well and incubated at 37°C for 1 h followed by being washed four times. 100 *μ*l per well of anti-mouse HRP-IgG, IgG1, IgG2a, or IgM was added and then incubated at 37°C for 1 h. After four times washing by PBST, TMB substrate was added to the 96-well plates with a volume of 100 *μ*l per well and incubated for 5 min at room temperature. 50 *μ*l of 2 M H_2_SO_4_ was used to stop the reaction. The values of OD_450_ were recorded.

### 2.13. Statistical Analysis

GraphPad Prism 5 software (GraphPad Software, USA) was used to analyze the results. The differences between the groups of mice were compared by one-way ANOVA. The values of *P* < 0.05 (∗) represented the statistically significant, and *P* < 0.01 (∗∗) represented the highly statistically significant between the experimental groups.

## 3. Results

### 3.1. Purification of the PS-Protein Conjugates

**Figure 2:**
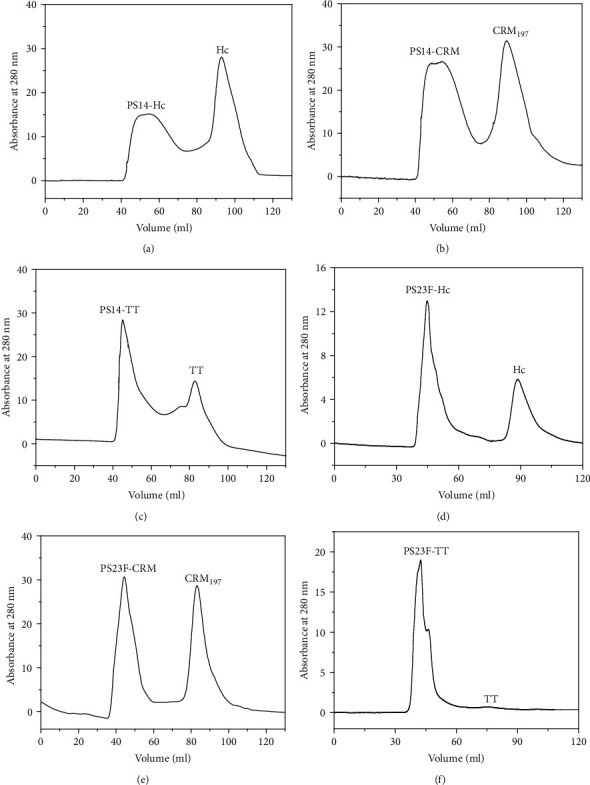
Purification of the conjugates. PS14-Hc (a), PS14-CRM (b), PS14-TT (c), PS23F-Hc (d), PS23F-CRM (e), and PS23F-TT (f) were purified by a Superdex 200 column (1.6 cm × 60 cm) at room temperature.

A Superdex 200 column (1.6 cm × 60 cm, GE) was used to purify the six conjugates based on SEC. As a result in Figure 2(a), PS14-Hc was first eluted as a broad symmetric peak at 55.3 ml, followed by a single peak of the unconjugated Hc. The reaction mixture containing PS14-CRM showed a chromatographic behavior similar to PS14-Hc, and the elution peak of PS14-CRM was at 52.4 ml (Figure 2(b)). In contrast, PS14-TT was first eluted as a significant peak at 45.2 ml, followed by a small peak of the unconjugated TT (Figure 2(c)). As a result in Figure 2(d), PS23F-Hc was eluted at the peak of 44.9 ml. Similarly, there also appeared two elution peaks of the reaction mixture of PS23F-CRM and the target elution peak was at 44.4 ml (Figure 2(e)). The reaction mixture containing PS23F-TT was eluted as a single peak at 42.4 ml (Figure 2(f)).

The elution peaks of the six conjugates appeared in the turn of PS23F-TT, PS23F-CRM, PS23F-Hc, PS14-TT, PS14-CRM, and PS14-Hc, which were proportional to the apparent molecular weights (MWs) of the conjugates. The PS23F-based conjugates were eluted more first than the PS14-based ones; due to that, PS23F showed higher MW than PS14. The CRM_197_-based conjugates were eluted earlier than the Hc-based ones and later than the TT-based ones. This result was due to that CRM_197_ displayed an Mw higher than Hc and lower than TT.

### 3.2. Molecular Radius Detection

The molecular radii of the carrier proteins and the conjugates were measured by dynamic light scattering. The molecular radii of TT, Hc, and CRM_197_ were 6.9 nm, 4.7 nm, and 5.2 nm, respectively. The molecular radii of PS14-TT and PS23F-TT were 11.9 nm and 14.3 nm, respectively. The molecular radii of PS14-Hc and PS23F-Hc were 10.3 nm and 12.4 nm, respectively. The molecular radii of PS14-CRM and PS23F-CRM were 10.8 nm and 12.9 nm, respectively. This analysis is consistent with the SEC result (Figure 2).

### 3.3. Quantitative Analysis

Free carrier proteins were not detected in the six conjugates. In contrast, the free PS14 ratios in PS14-Hc, PS14-TT, and PS14-CRM were 3.9%, 2.8%, and 3.3% (*w*/*w*), respectively. The free PS23F ratios in PS23F-Hc, PS23F-TT, and PS23F-CRM were 3.6%, 4.2%, and 3.0% (*w*/*w*), respectively. The PS14/protein ratios (*w*/*w*) of PS14-Hc, PS14-TT, and PS14-CRM were 0.56, 1.39, and 0.65, respectively. The PS23F/protein ratios (*w*/*w*) of PS23F-Hc, PS23F-TT, and PS23F-CRM were 0.62, 1.48, and 0.77, respectively. Due to the MWs of TT (140 kDa), Hc (50 kDa), and CRM_197_ (62 kDa), the PS14/protein and PS23F/protein ratios (mol/mol) of the six conjugates were comparable with each other.

### 3.4. Intrinsic Fluorescence Analysis

**Figure 3:**
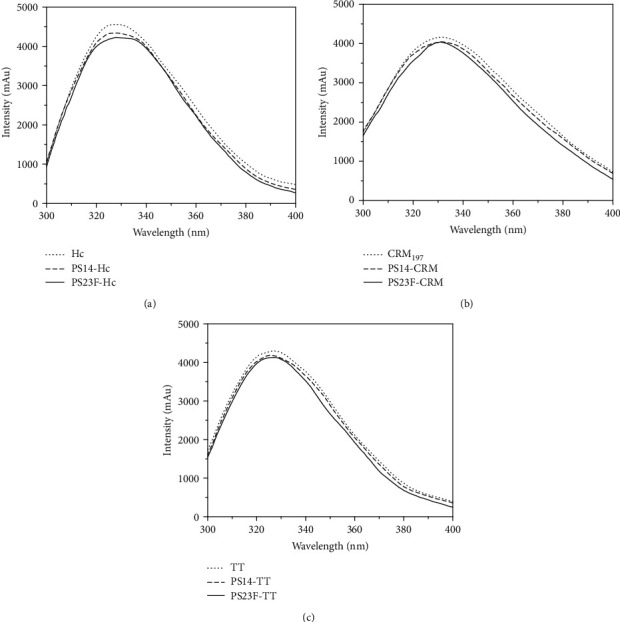
Fluorescence analysis of the conjugates. The intrinsic fluorescence emission spectra of the Hc-based conjugates (a), the CRM_197_-based conjugates (b), and the TT-based conjugates (c) were recorded from 300 to 400 nm.

Due to Trp and Tyr residues, proteins have intrinsic fluorescence. As a result in Figure 3(a), Hc showed a unimodal curve with a maximum wavelength of 328 nm. The emission fluorescence intensities of PS14-Hc and PS23F-Hc were slightly lower than that of Hc with an inapparent change of the maximum wavelength. As shown in Figure 3(b), CRM_197_ displayed a unimodal curve with a maximum wavelength of 332 nm. The emission fluorescence intensities of PS14-CRM and PS23F-CRM were comparable to that of CRM_197_, accompanied by no shift in the maximum wavelength. As a result in Figure 3(c), TT exhibited a unimodal curve with a maximum wavelength of 327 nm. The curve of PS14-TT or PS23F-TT was almost overlapped with that of TT. Thus, the conjugation of PS14 and PS23F did not fundamentally change the Trp and Tyr environment of the carrier proteins. In other words, the conformations of the carrier proteins did not change when binding with PS.

### 3.5. Secondary Structure Analysis

**Figure 4:**
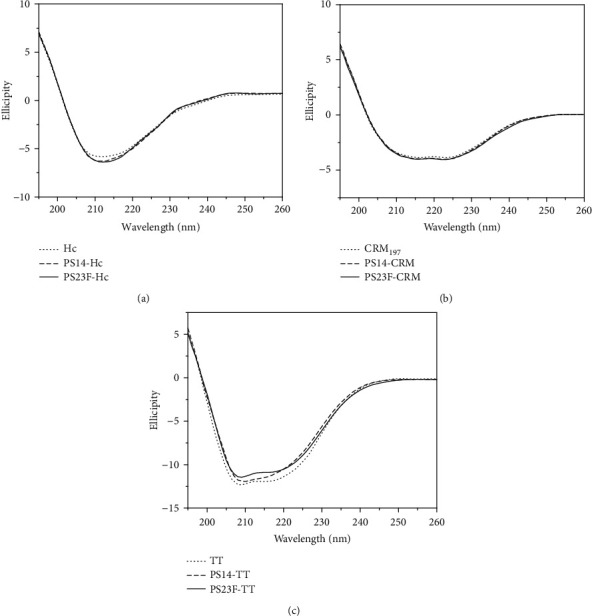
Circular dichroism analysis of the conjugates. The far-UV circular dichroism spectra of the Hc-based conjugates (a), the CRM_197_-based conjugates (b), and the TT-based conjugates (c) were recorded from 260 to 190 nm.

Far-UV circular dichroism (CD) spectroscopy was used to measure the secondary structures of the six conjugates. As a result in Figure 4, Hc, CRM_197_, and TT all showed typical CD spectra. As a result in Figure 4(a), the CD spectra of PS16-Hc or PS23F-Hc were almost overlapped on that of Hc. Similarly, the CD spectra of PS16-CRM and PS23F-CRM were essentially coincided with that of CRM_197_ (Figure 4(b)). The CD spectra of PS16-TT and PS23F-TT were close to that of TT (Figure 4(c)). Thus, the secondary structures of Hc, CRM_197_, and TT were not essentially altered upon conjugation with PS.

### 3.6. PS14-Specific Antibodies

**Figure 5:**
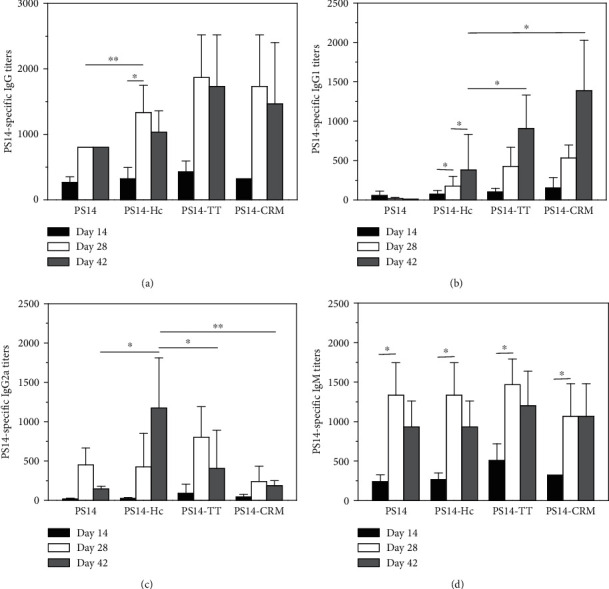
PS14-specific antibody titers elicited by the PS14-based conjugates. The measurements of PS14-specific IgG (a), IgG1 (b), IgG2a (c), and IgM (d) were carried out using ELISA. Blood samples after immunization on days 42 were obtained for antibody measurement. Each sample was measured three times. Bar represented mean ± S.D. from 6 mice per group.

ELISA assay was used to measure the PS14-specific antibody titers in mice. As a result in Figure 5(a), the IgG titers of PS14-specific antibodies in the four groups were all very low after the first vaccination (day 14). The PS14 group showed a 2.8-fold increase in the PS14-specific IgG titers after the second immunization (day 28) compared with the first immunization (~1 : 270). The third vaccination (day 42) could not further booster the IgG titers. However, the PS14-specific IgG titers of the PS14-Hc group showed a 4.2-fold increase after the second immunization (day 28), which was higher than those of the PS14 group (*P* < 0.01). The third vaccination could not further booster the IgG titers. The IgG titers of the PS14-TT (1 : 1.7 × 10^3^) and PS14-CRM groups (1 : 1.4 × 10^3^) were not significantly different from the PS14-Hc group on day 42 (1 : 1.1 × 10^3^, *P* > 0.05). Thus, Hc could act as a capable carrier protein to enhance the PS14-specific IgG titers like CRM_197_ and TT.

As shown in Figure 5(b), the four groups all showed low PS14-specific IgG1 titers after the first immunization (day 14). As a marker of the Th2 pathway, IgG1 titer of the PS14 group could hardly be detected after the second and third vaccination. In contrast, the PS14-Hc group got a 2.4-fold increase of the specific IgG1 titers after the second (~1 : 180) vaccination and a 5.2-fold increase of the specific IgG1 titers after the third (~1 : 380) vaccinations. The IgG1 titers of the PS14-TT (~1 : 900) and PS14-CRM groups (1 : 1.3 × 10^3^) were both higher than that of the PS14-Hc group on day 42 (~1 : 380, *P* < 0.05).

As a result in Figure 5(c), the PS14-specific IgG2a titers of the PS14 group were almost undetectable upon the three vaccinations. As a sign of the Th1 immune pathway, the IgG2a titers of the other three groups were undetectable upon initial vaccination and significantly increased upon the second and third vaccinations. In particular, the PS14-Hc group showed significantly higher IgG2a titers on day 42 (1 : 1.1 × 10^3^) than those of the PS14-TT group (~1 : 430) (*P* < 0.05) and PS14-CRM groups (~1 : 190) (*P* < 0.01). The ratios of IgG2a/IgG1 in the PS14-Hc, PS14-TT, and PS14-CRM groups on day 42 were 2.90, 0.48, and 0.15, respectively. Thus, the Th1/Th2 bias of the PS14-Hc group was significantly different from the PS14-TT and PS14-CRM groups. It is suggested that Hc could stimulate stronger cellular immunity than TT and CRM_197_ as a carrier protein.

As a result in Figure 5(d), the IgM titers in the four groups were low upon initial vaccination and significantly increased upon the second vaccination. The third vaccination could not further booster the IgG titers. Moreover, the PS14-specific IgM titers of the four groups were comparable to each other upon the three vaccinations. Besides, the IgG/IgM ratios of the PS14-Hc, PS14-TT, and PS14-CRM groups on day 42 were 1.1, 1.4, and 1.4, respectively. These ratios were higher than those at the first vaccination (1.0, 0.8, and 1.0). This result suggested that the conjugation of PS14 with the carrier proteins shifted more response from IgM to IgG.

### 3.7. PS23F-Specific Antibodies

**Figure 6:**
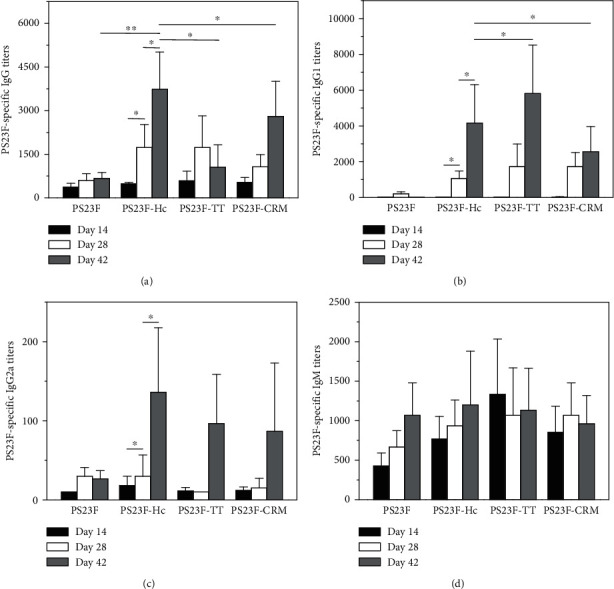
PS23F-specific antibody titers elicited by the PS23F-based conjugates. The measurements of PS23F-specific IgG (a), IgG1 (b), IgG2a (c), and IgM (d) were carried out using ELISA. Blood samples after immunization on days 42 were obtained for antibody measurement. Each sample was measured three times. Bar represented mean ± S.D. from 6 mice per group.

As a result in Figure 6(a), all groups showed low IgG titers of the PS23F-specific antibodies after the first immunization (day 14). The PS23F group did not booster the PS23F-specific IgG titers after the second and third immunizations compared with the initial vaccination of PS23F. Compared to the first immunization of PS23F-Hc (~1 : 480), the IgG titers of the PS23F-Hc group showed a 3.6-fold increase upon the second immunization and a 7.8-fold increase upon the third immunization. Moreover, the PS23F-specific IgG titers of the PS23F-Hc group were higher than those of the PS23F group (*P* < 0.01). Thus, the immunogenicity of PS23F-Hc was substantial after two doses with a positive booster response observed after the second dose. The PS23F-specific IgG titers of the PS23F-TT (1 : 1.0 × 10^3^) and PS23F-CRM groups (1 : 2.8 × 10^3^) on day 42 were both lower than those of the PS23F-Hc group (1 : 3.7 × 10^3^) (*P* < 0.05). Thus, Hc showed a more powerful carrier effect than TT and CRM_197_ on the PS23F-specific IgG titers.

As a result in Figure 6(b), all the groups showed low IgG1 titers of the PS23F-specific antibodies after the initial vaccination. The second and third vaccinations could significantly increase the IgG1 titers of the three conjugate groups (*P* < 0.05). Particularly, the IgG1 titers of the PS23F-Hc group (4.2 × 10^3^) were lower than those of the PS23F-TT group (5.8 × 10^3^) and higher than those of the PS23F-CRM groups (2.5 × 10^3^) (*P* > 0.05) on day 42.

As a result in Figure 6(c), the PS23F-specific IgG2a titers of the PS23F group were undetectably low after all three vaccinations. The Th1-type IgG2a titers of the other three groups were undetectable upon initial vaccination and slightly increased upon the second (*P* < 0.05) and third vaccinations (*P* < 0.05). Moreover, the IgG2a titers of the three groups on the third vaccination did not display a significant difference (*P* > 0.05). The ratios of IgG2a/IgG1 in the PS23F-Hc, PS23F-TT, and PS23F-CRM groups on day 42 were 0.03, 0.02, and 0.03, respectively. Thus, the PS23F-specific antibody response of the three groups was predominantly in the form of IgG1.

As a result in Figure 6(d), the IgM titers of the four groups were low upon initial vaccination. The second and third vaccinations could not significantly booster the IgG titers. Moreover, the PS23F-specific IgM titers of the four groups were comparable to each other upon the three vaccinations. Besides, the IgG/IgM ratios of the PS23F-Hc, PS23F-TT, and PS23F-CRM groups on the third vaccination were 3.1, 0.9, and 2.9, respectively. These ratios were higher than those at the first vaccination (0.6, 0.5, and 0.6). This result suggested that conjugation of the carrier proteins changed the response of IgM to IgG, which was a typical reaction of the conjugate vaccines.

## 4. Discussion

Hc was a safe, effective, and low-cost protein with robust immunogenic potency. Pneumococcal serogroups 14 and 23F were the major causes of invasive pneumococcal disease in children and older persons. The three carrier proteins (Hc, TT, and CRM_197_) were covalently conjugated with PS14 and PS23F in the present study. The six conjugates were used to investigate the carrier effect of Hc on the immunogenicity of PS14 and PS23F.

As T cell-independent antigens, PS14 and PS23F both have some repeating B cell epitopes. The T-helper cell epitopes of a carrier protein can activate the protein-specific CD4^+^ T cells, which may provide help to B cells in the internalization and process of the conjugate and presentation of peptides in MHC class II molecules [26]. The PS-specific B cells can induce its differentiation towards plasma or memory cells and elicit significant intensity of PS-specific antibody response [27]. Acquired protective immunity to *S. pneumonia* mainly due to the PS-specific antibodies naturally obtained against invasive pneumococcal diseases. Thus, the PS-specific antibody response of the conjugates was investigated in this study.

The conjugate vaccines were formed by PS, and the carrier proteins covalently conjugated. Several factors may affect the PS-specific antibody titers of the conjugates, such as the conjugation method, immunization protocol, carrier protein, PS antigen, and PS/protein molar ratio. The conjugation method, immunization protocol, and PS antigen were identical for the six conjugates. The PS14- and PS23F-based conjugates showed comparable PS/protein molar ratios. Thus, the carrier protein was the major differential factor of the conjugate vaccines.

In the present study, PS14-Hc and PS23F-Hc were demonstrated to potentiate a robust PS-specific antibody response at the levels comparable to the TT- and CRM_197_-based controls. Hc possessed multiple T-helper cell epitopes and exhibited a practical carrier effect on the immunogenicity of the conjugate vaccine, given the high levels of PS-specific antibodies that were measured. This result indicated a sufficient potency of Hc as a carrier protein. The PS14-specific IgG response after three doses was higher than the PS14-specific IgM response. A similar pattern was observed for PS23F-specific immune reactions. This result indicated that the two antigens were changed to T cell-dependent antigens by Hc.

Different from the protein vaccine, the antibody titers of the specific polysaccharide induced by the conjugate vaccine in animals do not seem high. Zhang et al. studied the immunogenicity of the 13 valent Streptococcus pneumoniae polysaccharide conjugate vaccine without adjuvants in rhesus monkeys. The results showed that the antibody titer for different polysaccharide types was 1 : 95.1 ~ 1 : 538.2 [28]; Shen et al. studied the antibody titers of NIH mice immunized intraperitoneally with Streptococcus pneumoniae capsular polysaccharide-tetanus toxoid conjugate vaccine for three times. The results showed that the antibody titers against 18C and 23F polysaccharide were 1 : 373.29 and 1 : 422.24, respectively [29]. In our study, the antibody titers of 14 and 23F polysaccharide in mice immunized with Hc conjugate vaccine three times can reach 1 : 1100 and 1 : 3700, which is much higher than that reported in previous literatures. It is speculated that the conjugate vaccines have strong immunogenicities and good protective activities.

TT and CRM197 are the typical carrier proteins for meningococcal and pneumococcal conjugate vaccines. Fluorescence spectroscopy data confirmed that Hc, CRM_197_, and TT all assumed mostly no conformational changes after conjugation with PS. In the population with preexisting immunity of TT or CRM_197_, the undesirable antibodies can decrease the effectiveness of the conjugate vaccines with carrier protein TT or CRM_197_. Thus, Hc is an ideal candidate carrier to avoid this problem.

## 5. Conclusion

In summary, conjugation with Hc significantly increased the immunogenicity of pneumococcal PS14 and PS23F. The carrier effect of Hc was comparable to TT and CRM_197_. Thus, Hc could act as a capable carrier protein for the development of capsular PS conjugate vaccines to prevent encapsulated bacterial infection. This study lends credence to the notion that it is vital to select an appropriate carrier to enable the epitopes of PS for the induction of proper antibody response.

## Data Availability

All the data are available from Dr. Rui Yu yurui1102@139.com upon request.

## Additional Points

*Highlights*. This is the first report about tetanus toxin native C-fragment (Hc), which is safe, low-cost, highly immunogenic, and easy to purify, used as the carrier protein of polysaccharide binding vaccine.
